# Oligometastatic Prostate Cancer Treated with Metastasis-Directed Therapy Guided by Positron Emission Tomography: Does the Tracer Matter?

**DOI:** 10.3390/cancers15010323

**Published:** 2023-01-03

**Authors:** Francesco Lanfranchi, Liliana Belgioia, Michela Marcenaro, Elisa Zanardi, Giorgia Timon, Mattia Riondato, Veronica Giasotto, Jeries Paolo Zawaideh, Laura Tomasello, Guglielmo Mantica, Nataniele Piol, Marco Borghesi, Paolo Traverso, Camilla Satragno, Daniele Panarello, Claudio Scaffidi, Andrea Romagnoli, Sara Elena Rebuzzi, Angela Coco, Bruno Spina, Silvia Morbelli, Gianmario Sambuceti, Carlo Terrone, Salvina Barra, Giuseppe Fornarini, Matteo Bauckneht

**Affiliations:** 1 Department of Health Sciences (DISSAL), University of Genoa, 16132 Genova, Italy; 2 Radiation Oncology, IRCCS Ospedale Policlinico San Martino, 16132 Genova, Italy; 3 Academic Unit of Medical Oncology, IRCCS Ospedale Policlinico San Martino, 16132 Genova, Italy; 4 Nuclear Medicine, IRCCS Ospedale Policlinico San Martino, 16132 Genova, Italy; 5 Department of Radiology, IRCCS Ospedale Policlinico San Martino, 16132 Genova, Italy; 6 Department of Urology, IRCCS Policlinico San Martino, 16132 Genova, Italy; 7 Pathology Unit, IRCCS Ospedale Policlinico San Martino, 16132 Genova, Italy; 8 Department of Surgical and Diagnostic Integrated Sciences, University of Genova, 16132 Genova, Italy; 9 Department of Experimental Medicine (DIMES), University of Genoa, 16132 Genova, Italy; 10 Medical Oncology Unit, Ospedale San Paolo, 17100 Savona, Italy; 11 Department of Internal Medicine and Medical Specialities (DiMI), University of Genoa, 16132 Genova, Italy; 12 Medical Oncology Unit 1, IRCCS Ospedale Policlinico San Martino, 16132 Genova, Italy

**Keywords:** PET/CT, [68Ga]Ga-PSMA-11, [18F]F-Fluorocholine, prostate cancer, metastasis-directed therapy, SBRT

## Abstract

**Simple Summary:**

Metastatic disease is the leading cause of morbidity and mortality in prostate cancer. Current therapeutic strategies mostly target cancer cells with important systemic effects injuring normal cells and healthy tissues. In prostate cancer patients with less than five metastases (called “oligometastatic”), this could be circumvented by implementing metastases-directed therapies (e.g., stereotactic radiotherapy), potentially avoiding systemic toxicity. In this setting, more accurate disease staging will likely result in more patients receiving the appropriate treatment, with expected better oncological results. On this basis, we verified the impact of two different radiotracers for Positron Emission Tomography/Computed Tomography imaging used as the guide for metastases-directed therapy on the oncological outcome in a retrospective sample of prostate cancer patients having less than five distant metastases. Obtained data showed that using next-generation imaging with [68Ga]Ga-prostate-specific membrane antigen-11 Positron Emission Tomography/Computed Tomography might favourably impact the oncological outcome of oligometastatic prostate cancer patients treated with metastases-directed therapy.

**Abstract:**

The superior diagnostic accuracy of [68Ga]Ga-prostate-specific membrane antigen-11 (PSMA) ([68Ga]Ga-PSMA-11) compared to [18F]F-Fluorocholine Positron Emission Tomography/Computed Tomography (PET/CT) in Prostate Cancer (PCa) is established. However, it is currently unclear if the added diagnostic accuracy actually translates into improved clinical outcomes in oligometastatic PCa patients treated with [68Ga]Ga-PSMA-11 PET-guided metastasis-directed therapy (MDT). The present study aimed to assess the impact of these two imaging techniques on Progression-Free Survival (PFS) in a real-world sample of oligometastatic PCa patients submitted to PET-guided MDT. Thirty-seven oligometastatic PCa patients treated with PET-guided MDT were retrospectively enrolled. MDT was guided by [18F]F-Fluorocholine PET/CT in eleven patients and by [68Ga]Ga-PSMA-11 PET/CT in twenty-six. Progression was defined as biochemical recurrence (BR), radiological progression at subsequent PET/CT imaging, clinical progression, androgen deprivation therapy initiation, or death. Clinical and imaging parameters were assessed as predictors of PFS. [18F]F-Fluorocholine PET-guided MDT was associated with significantly lower PFS compared to the [68Ga]Ga-PSMA-11 group (median PFS, mPFS 15.47 months, 95% CI: 4.13–38.00 vs. 40.93 months, 95% CI: 40.93–40.93, respectively; *p* < 0.05). Coherently, the radiotracer used for PET-guided MDT resulted in predictive PFS at the univariate analysis, as well as the castration-resistant status at the time of MDT and the PSA nadir after MDT. However, in the multivariate analysis, castration resistance and PSA nadir after MDT remained the sole independent predictors of PFS. In conclusion, in the present proof-of-concept study, [68Ga]Ga-PSMA-11 provided higher PFS rates than [18F]F-Fluorocholine imaging in oligometastatic PCa patients receiving PET-guided MDT. Although preliminary, this finding suggests that enlarging the “tip of the iceberg”, by detecting a major proportion of the submerged disease thanks to next-generation imaging may favourably impact the oncological outcome of oligometastatic PCa treated with MDT.

## 1. Introduction

The primary management of metastatic prostate cancer (PCa) is androgen deprivation therapy (ADT). However, even though many patients can undergo ADT for years before progression or failure, it rarely eradicates metastatic disease permanently [1]. This is also true when ADT is combined with newer androgen receptor signalling inhibitors [2,3,4,5,6]. Additionally, ADT is associated with relevant side effects, such as fatigue, mood alterations, and metabolic disorders [6]. Thus, radical-intent treatments such as metastasis-directed therapy (MDT) through stereotactic body radiotherapy (SBRT) should be considered when metastases are limited in number and location. In fact, deferring ADT initiation represents a considerable clinical advance for PCa patients, and systemic adverse events could be avoided.

The role of MDT has been widely explored in oligometastatic PCa. Patients treated with MDT presented a significantly prolonged time to initiation of ADT [7,8,9]. Moreover, MDT provided local control and showed good tolerability, favourably impacting the long-term oncological outcome in both de novo and metachronous low-volume metastatic PCa [7,9]. However, a crucial issue of MDT is that the imaging technique used to define lesions should be accurate in detecting metastases. It is presumed that the more precise the disease identification, the higher percentage of patients who will receive the appropriate MDT treatment, with expected better oncological results. In the 2017 Advanced Prostate Cancer Consensus Conference meeting, oligometastatic PCa was defined based on standard imaging methods, including bone scintigraphy, contrast-enhanced computed tomography (CT), and Magnetic Resonance Imaging [10]. However, although currently recommended, these techniques have poor diagnostic accuracy, underestimating the actual number of metastatic sites [11].

In recent years, Positron Emission Tomography/Computed Tomography (PET/CT) has dramatically improved the detection of PCa recurrences compared to conventional imaging [12]. It is presumed that detecting a greater proportion of micro-metastatic disease thanks to next-generation imaging may favourably impact the oncological outcome of oligometastatic PCa patients receiving MDT. However, to date, no consensus regarding the use of modern imaging methods in PCa exists, as no clinical trials that evaluate the benefits of treating oligometastases identified with these methods are available yet.

Similarly, the hypothetical advantage of displaying cell membrane phospholipid synthesis as an index of cell growth (with radio-labelled choline) or targeting the expression of Prostate-Specific Membrane Antigen (PSMA) on PCa tumour cells through PET/CT still needs to be verified in this clinical setting. For several years, [18F]F-Fluorocholine and [11C]C-choline PET/CT have been recommended by international guidelines as the gold-standard approach for PCa restaging in the presence of biochemical relapse [13]. Recently, [68Ga]Ga- or [18F]F-labelled PSMA tracers became the imaging tool of choice according to the last international guidelines, being able to provide higher sensitivity even in the presence of low Prostate-Specific Antigen (PSA) serum levels (0.2–1 ng/mL) [14]. Thanks to the higher sensitivity, it can be hypothesised that the early MDT intervention on PSMA-expressing oligometastases might favourably impact the clinical history of oligometastatic PCa patients, while the consolidation of the macroscopic choline-positive disease may simply reset the clock on time to detectable metastases, allowing micrometastatic disease to spread unchecked until reaching sufficient size to become clinically evident.

On these bases, the present study aimed to preliminarily compare the impact of these two tracers on the oncological outcome in a retrospective real-world sample of oligometastatic PCa patients subjected to PET-guided MDT.

## 2. Materials and Methods

### 2.1. Study Population and Clinical Data Collection

We retrospectively enrolled consecutive oligometastatic PCa patients submitted to PET-guided MDT between June 2017 and December 2021 at our institution. Patients were informed that their clinical and imaging data could have been used for retrospective research purposes and gave their written consent for usage and publication in an anonymized form. The study was conducted according to the guidelines of the Declaration of Helsinki. The local ethical committee approved the study (Regional Ethical Committee of Liguria-registration number 343/2019).

Inclusion criteria were: (i) histologically confirmed diagnosis of PCa; (ii) pelvic or extra-regional nodal relapse (M1a), bone metastases (M1b) detected by either choline or PSMA PET/CT imaging; (iii) the presence of no more than five lesions at PET/CT imaging preceding MDT administration (oligometastatic PCa); (iv) upfront MDT delivered through SBRT +/− systemic therapy. The sole exclusion criterion was the unavailability of the subsequent clinical follow-up. This process led to the identification of 37 oligometastatic PCa patients (mean age 73.7 ± 7.6 years old).

The following demographic and clinical variables were collected: Gleason score, International Society of Urological Pathology (ISUP) grade at diagnosis, primary radical treatment, salvage radiation therapy, the time interval between diagnosis and oligometastatic state, age at MDT, number of CRPC patients at MDT, the addition of systemic treatment to MDT, number and sites of metastases treated with MDT, MDT total dose and biologically effective dose, PSA nadir after MDT, time to PSA nadir, and the number of patients which experienced progression at follow-up.

### 2.2. PET/CT Images Acquisition and Analysis

[18F]F-Fluorocholine and [68Ga]Ga-PSMA-11 PET/CT were performed according to current guidelines [15,16].

Integrated PET/CT scanners using either Hirez-Biograph 16 (Siemens Medical Solutions, Munich, Germany) or Biograph mCT Flow (Siemens Medical Solutions, Munich, Germany) were used to perform a whole-body (skull vertex to the upper thighs) PET acquisition in the three-dimensional mode (emission time: 2 min per bed position with an axial field-of-view of 15.6 cm). A low-dose CT was performed for attenuation correction and anatomical allocation. No diagnostic contrast-enhanced CT scans were performed. Reconstruction was performed with an ordered subset expectation maximization algorithm with four iterations per eight subsets. Images were corrected for random coincidences and scatter.

Images were analyzed using Syngo.via software (Siemens Medical Solutions, Munich, Germany) by two experienced nuclear medicine physicians in consensus. For each lesion identified on transaxial images, a volume of interest (VOI) was drawn with an isocounter threshold based on 40% of the SUVmax. Maximum standardized uptake values (SUVmax), metabolic tumor volume (MTV, calculated as the sum of all lesions), and total lesion glycolysis (TLG, calculated as the sum of the products between MTV and the corresponding SUVmean) were collected.

### 2.3. PET-Guided MDT and Clinical Follow-Up

As established by our institutional protocol, patients were managed according to current International Guidelines [17] and good clinical practice recommendations. Before MDT, all patients underwent a CT-based treatment planning with a 2-mm slice thickness in the supine position. Gross Tumor Volume (GTV) was delineated using all the available morphological and PET/CT imaging information. An isotropic margin of 3 mm was added to GTV to create the Clinical Target Volume (CTV) for bone lesions. A planning target volume (PTV) was created around the CTV using isotropic or anisotropic 2-mm margin. All patients received intensity-modulated radiotherapy (IMRT) or Volumetric Modulated Arc Therapy (VMAT) with Image-guided radiotherapy. Collected MDT parameters included the total dose administered, the number of fractions, the biologically effective dose, and the eventual administration of ADT. The radiation schedule most used was 35 Gy in five fractions.

Following MDT, patients entered a short-term clinical follow-up according to our institutional protocol, including clinical evaluation and PSA blood test every 3 to 4 months. [18F]F-Fluorocholine or [68Ga]Ga-PSMA-11 PET/CT restaging was performed using the corresponding MDT-guiding radiotracer in case of detectable PSA or biochemical progression post-MDT, defined according to PC Working Group 3 [18]. In the case of oligo-progression after MDT, further courses of MDT were generally proposed if less than five new lesions were detected by PET/CT imaging outside the previously irradiated field. Systemic treatments were administered in case of a polymetastatic conversion of the disease. Patients with disease progression were followed up for survival status (long-term follow-up).

### 2.4. Statistical Analysis

The primary endpoint of the study was Progression-Free Survival (PFS) in oligometastatic PCa patients submitted to [68Ga]Ga-PSMA-11 versus [18F]F-Fluorocholine guided MDT. Progression was defined as a composite endpoint as previously described [8,9]. Briefly, any of the following conditions were considered as a progression: (i) PSA rise of at least 2 ng/dL and 25% above nadir; (ii) concern for radiologic progression by PET/CT; (iii) symptomatic progression of disease; (iv) initiation of ADT or systemic treatment change for any reason; (v) death.

All patients’ variables were assessed with the Kolmogorov-Smirnov to evaluate data distribution. Continuous variables were compared with the *t*-test, while categorical variables were tested with the chi-square test. Cox regression analysis was performed assuming PFS as the dependent variable. Univariate analyses were performed to correlate PFS with the available clinical and imaging parameters. All variables with a *p* < 0.100 entered the multivariate analysis. Hazard ratios (HR) for Cox regression models were reported with a 95% confidence interval (CI) and *p*-value. The Kaplan-Meier method (log-rank test) was also applied once continuous variables were dichotomized. Statistical significance was set at *p* < 0.050. Data analysis was conducted by using SPSS^®^ software version 26 (IBM Corp., Armonk, NY, US).

## 3. Results

### 3.1. Patients’ Clinical Characteristics, PET-Guided MDT, and Clinical Follow-Up

The clinical characteristics of the entire sample are summarized in Table 1. Eleven patients (29.8%) underwent [18F]F-Fluorocholine PET/CT, while twenty-six (70.2%) were submitted to [68Ga]Ga-PSMA-11 PET/CT for restaging purposes after primary treatment.

The two subgroups were balanced concerning baseline bioptical ISUP grade, primary treatment, previous salvage radiotherapy, and time from PCa diagnosis to oligometastatic disease onset (*p* = ns for all) (Table 1). At the time of PET oligorecurrence detection, age and CRPC status were superimposable between the two groups (*p* = ns for both), and PSA serum levels were not significantly different between the two subgroups of patients (4.19 ± 5.38 vs. 2.50 ± 6.74, *p* = 0.468, respectively). PET/CT imaging identified 48 lesions, including 37 lymph node metastases (77%) and 11 bone lesions (23%), while no visceral metastases were detected. Most recruited patients had one metastatic site (100% for patients receiving [18F]F-Fluorocholine-guided MDT), and the percentage of nodal and bone metastases were balanced between the two study groups (Table 1). No other significant differences between the two subgroups were detected (Table 1). In particular, MDT total dose and biologically effective dose were not significantly different between the two groups of patients (*p* = ns for both). Notably, the concurrent administration of medical therapy in addition to MDT was equally balanced between the two subgroups (91% vs. 73% in the [18F]F-Fluorocholine and the [68Ga]Ga-PSMA-11 subgroups, respectively, *p* = ns).

**Figure 1:**
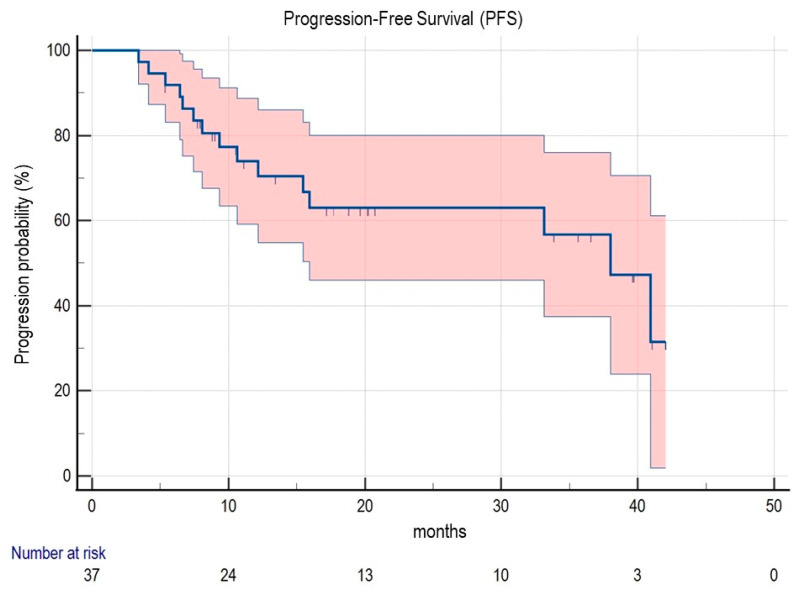
Kaplan-Meier curve for PFS in the whole sample. Abbreviations: PFS, progression-free survival.

Considering the entire sample, the median time interval from PET-guided MDT to the PSA nadir was 4 months (5 months in the [68Ga]Ga-PSMA-11 subgroup vs. 4 months in the [18F]F-Fluorocholine subgroup; *p* = ns). However, the PSA level at the nadir was significantly lower in patients submitted to [68Ga]Ga-PSMA-11 PET/CT guided MDT (Table 1, *p* = 0.018). After MDT, patients were clinically and biochemically followed up for a median interval of 20.48 months (range 5.37–60.13), and the median PFS (mPFS) was 40.93 months (95% CI 15.93–40.93) (Figure 1).

Of the 15 (40.5%) patients presenting biochemical relapse after MDT, the number of progressions was higher in the [18F]F-Fluorocholine (9/11, 81.8%) compared to the [68Ga]Ga-PSMA-11 subgroup (6/26, 23.1%; *p* = 0.001). Among relapsed patients, 14 (93.3%) were restaged to PET/CT imaging (which confirmed the progression in all cases), and 1 died (6.7%, belonging to the [18F]F-Fluorocholine subgroup). In all cases, PET/CT restaging was performed using the same tracer previously driving MDT. In 5 cases, the persistence of the oligometastatic status was shown by the restaging PET/CT (2/5 in the [18F]F-Fluorocholine subgroup and 3/5 in the [68Ga]Ga-PSMA-11 subgroup), thus guiding a second-line of MDT. Second-line MDT was performed in the same sites of initial treatment in 3 cases (2/3 in the [18F]F-Fluorocholine subgroup and 1/3 in the [68Ga]Ga-PSMA-11 subgroup).

### 3.2. Predictors of Clinical Outcome

**Figure 2:**
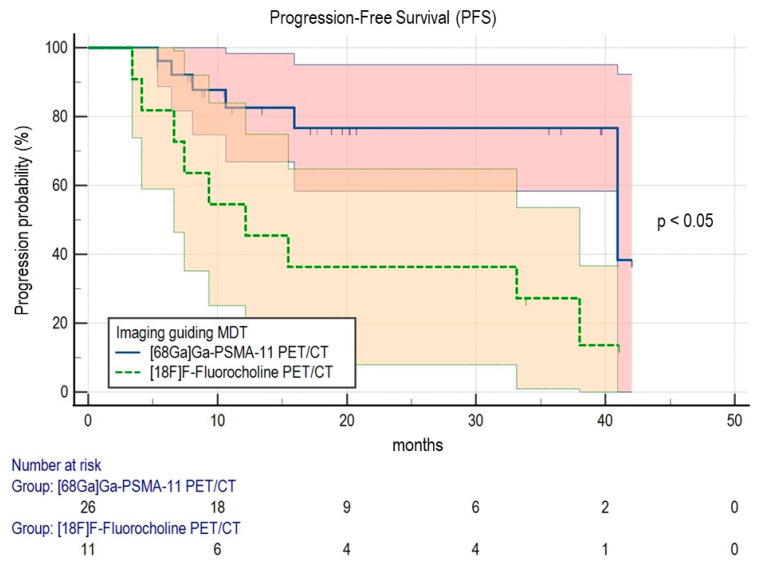
Kaplan-Meier curves for PFS according to the PET/CT radiotracer adopted to guide MDT. The comparison was performed with the Log Rank test. Abbreviations: MDT, metastasis-directed therapy; PET/CT, positron emission tomography/computed tomography; PFS, progression-free survival.

In the univariate analysis (Table 2), a significant association with lower PFS was reached by CRPC status at the time of MDT (HR 6.238, 95% CI 1.939–20.073; *p* = 0.002), PSA nadir after MDT (HR 2.979, 95% CI 1.882–4.717; *p* < 0.001) and by the use of [18F]F-Fluorocholine PET/CT imaging as a guide for MDT (HR 3.813, 95% CI 1.351–10.760; *p* = 0.011). Kaplan-Meier analysis was performed to explore better the impact of the two PET/CT radiotracers on PFS (Figure 2). In line with the previous result, patients submitted to [18F]F-Fluorocholine PET/CT showed a significantly lower mPFS compared to the [68Ga]Ga-PSMA-11 subgroup (15.47 months, 95% CI 4.13–38.00 vs. 40.93 months, 95% CI 40.93–40.93, respectively, *p* = 0.047). CRPC status and PSA nadir after MDT turned out to be independent predictors of PFS at the multivariate analysis (*p* = 0.024 and *p* < 0.001, respectively, Table 2), while no other clinical or imaging parameters reached significance.

## 4. Discussion

Although no consensus exists on the exact number of lesions shaping the oligometastatic state [19,20], the rationale underlying its treatment with MDT is that not only the primary tumor but also distant metastases can be the source of new tumor spread [21]. SBRT does not represent the unique available MDT [22,23]. However, it is currently the most used approach and is growing, as a four-fold increase in its utilization has been observed in the last decade [24].

In the phase II STOMP trial, MDT significantly prolonged the time to initiation of ADT in PCa patients with one to three metastases compared to observation [7]. Besides postponing ADT, the ablation of the oligometastatic foci through MDT also provided local control, showed good tolerability, and favourably impacted the long-term oncological outcome in both de novo and metachronous low-volume metastatic PCa [7]. In the ORIOLE phase II study, 54 oligometastatic PCa patients in a washout from ADT within 6 months of enrolment were randomized to MDT or observation [8]. The 6-month progression occurred in 19% and 61% of patients, respectively. The sustained clinical benefit of MDT was recently confirmed by pooling the STOMP and ORIOLE study cohorts [9]. On the one side, these data raised attention on this topic, promoting the development of further clinical trials addressing the oligometastatic phase of PCa. On the other hand, they pose several open issues for the future, including identifying the appropriate imaging guide for MDT.

The use of PET/CT imaging as the guide for MDT is supposed to impact its effectiveness in oligometastatic PCa, avoiding undertreatment and prolonging the long-term oncological outcome. However, PET/CT can be performed with many radiotracers for patients with PCa in the clinical setting. Generally, the choice of the preferred radiotracer may depend on its availability, the associated costs, the healthcare system’s reimbursement regulations, and the centre’s experience in reading and interpreting imaging results. On these bases, it is necessary to verify the eventual superiority of a specific tracer compared to the others. Even though a large amount of data demonstrated the superior diagnostic accuracy of PSMA-targeted radiotracers compared to either [11C]C-choline or [18F]F-Fluorocholine in the restaging of PCa with low PSA circulating levels [25], no clear superiority has been identified for patients with higher PSA serum levels [25] or with late-stage castration-resistant disease [26]. Moreover, growing data shows that PSMA expression can be heterogeneous in patients treated with systemic therapies [27,28]. These sources of heterogeneity may result in dissimilar response rates to MDT if guided by PSMA tracers. On these bases, the favourable impact of PSMA-targeted tracers compared to radio-labelled choline in the oligometastatic setting candidate to MDT is not a foregone conclusion. Accordingly, comparing it with the other most widely used PET tracer for PCa imaging as the guide for MDT, collecting data from the real-world setting represents an unmet clinical need.

In the present study, [68Ga]Ga-PSMA-11 PET/CT provided higher PFS rates than [18F]F-Fluorocholine PET/CT imaging in oligometastatic PCa patients receiving PET-guided MDT. Coherently, the PSA nadir following MDT was significantly lower in patients submitted to MDT under the guidance of [68Ga]Ga-PSMA-11, indicating a more pronounced biochemical response after therapy. This is relevant, as in our multivariable analysis, the PSA nadir appeared as the most potent prognosticator after MDT. Conversely to the ORIOLE and the STOMP trials [7,8,9], we collected data from PCa patients submitted to either exclusive MDT or MDT plus systemic treatment. This choice prevented the capability to include ADT-free survival as a clinical endpoint of our study. However, given that the concurrent administration of systemic therapy was equally distributed between the two subgroups of patients, we can assume that the choice of the radiotracer for PET-guided MDT is prognostically relevant regardless of the concurrent systemic therapy. However, due to the lack of statistical power, we could not perform dedicated subanalyses, which might identify eventual differences in specific patient groups. Similarly, given the real-world design, we retrospectively recruited consecutive hormone-sensitive and castration-resistant PCa patients, which were equally distributed between the two subgroups of patients. As expected, the CRPC state was a significant prognosticator in both the univariate and multivariate analyses. However, we could not perform dedicated subanalyses on CRPC patients due to the low statistical power.

Mazzola et al. recently published a retrospective study with a similar design [29]. The authors focused on the setting of an oligorecurrent hormone-sensitive PCa, showing that [68Ga]Ga-PSMA-11 PET/CT guided MDT with stereotactic radiotherapy related to an advantage in terms of ADT-free survival compared to [18F]F-Fluorocholine PET/CT. Interestingly, the Mazzola study observed no significant difference in ADT-free survival and PFS in patients with PSA levels >0.5 ng/mL in both groups [29]. Although our study did not replicate this finding, it still needs to be addressed as it might suggest that the advantage of [68Ga]Ga-PSMA-11 PET/CT in terms of oncological outcome might be heterogeneous considering the baseline PSA levels.

Of note, although supported by preliminary evidence, the added value of [68Ga]Ga-PSMA-11 PET/CT in this clinical setting has already been partially accepted by the clinical audience. In 2019, a paper by RADAR (Radiographic Assessments for Detection of Advanced Recurrence) III Group was published, with a strong recommendation for the use of next-generation imaging in patients with biochemically recurrent prostate cancer [30]. Shortly after, a survey conducted by AIRO (Italian Association of Radiotherapy and Clinical Oncology) genitourinary group focused on the clinical practice of radiation oncologists, showing the tendency to adopt molecular imaging both before and after MDT [31]. Using the Delphi consensus methodology, the European Society for Radiotherapy and Oncology (ESTRO) Guidelines Committee recommendations were recently published to standardize radiotherapy treatment of oligometastatic PCa patients [17]. Involved panellists reached a consensus in considering PSMA-targeted PET/CT the preferred staging and restaging imaging modality for oligometastatic, oligorecurrent, and oligoprogressive PCa [17]. Considering our data, this choice sounds reasonable. However, it should be noted that the retrospective evidence of a favourable impact on the clinical outcome is insufficient to support a change in clinical practice. As stated above, larger prospective studies might allow addressing several open issues, including the advantage of [68Ga]Ga-PSMA-11 PET/CT as the guide for MDT alone vs. MDT + systemic therapy, for hormone-sensitive vs. castration-resistant PCa patients and PCa patients with different PSA serum levels at baseline. Moreover, systematically introducing a highly sensitive next-generation imaging tool as the guide for MDT may lead to the so-called “stage migration phenomenon”, causing the upstaging of a subgroup of PCa patients from the oligometastatic state to the disseminated condition [32]. This may prevent the prescription of MDT in the subgroup of patients affected by the disseminated disease, in whom the unique therapeutic alternative would be the anticipation of systemic therapy. Thus, the hypothetical advantage in terms of the clinical outcome of next-generation imaging-guided MDT needs to be counterbalanced by the long-term effects of systemic treatment anticipation in this subgroup of patients. Improving the statistical power by recruiting a larger number of oligometastatic patients submitted to PET-guided MDT may not be sufficient to address this open issue and thus support the translation of [68Ga]Ga-PSMA-11 PET/CT-guided MDT into the clinical practice. Prospective and randomized trials are mandatory to address the consequences of the stage migration phenomenon provided by next-generation imaging [33].

## 5. Limitations

The small number of patients in our study is the major limitation of this work. Moreover, among 37 patients recruited, patients who submitted to [68Ga]Ga-PSMA-11 PET/CT-guided MDT were more numerous with respect to the [18F]F-Fluorocholine subgroup (26 and 11 patients, respectively). Thus, further studies are needed to confirm our findings in larger and more homogeneous samples of oligometastatic PCa patients. Second, the real-world design of this study led to the inclusion of both hormone-sensitive and CRPC patients. Again, the limited number of patients did not allow for performing dedicated subanalyses comparing these two subgroups. Finally, it is known that ADT can negatively affect the quality of life in PCa patients [6] and that MDT could avoid systemic adverse events associated with ADT with good tolerability [7,8,9]. On this basis, it might be of interest to verify any difference in MDT guided by [18F]F-Fluorocholine or [68Ga]Ga-PSMA-11 in postponing ADT and preserving the quality of life. Due to the retrospective nature of the present study, we could not address this relevant point, and thus further studies are needed.

## 6. Conclusions

In conclusion, the present data suggest that enlarging the “tip of the iceberg” by detecting a major proportion of the submerged disease thanks to next-generation imaging may favourably impact the oncological outcome of oligometastatic PCa patients treated with MDT. Further, prospective studies with randomized designs are still needed to support the clinical adoption of [68Ga]Ga-PSMA-11 PET/CT-guided MDT.

## Figures and Tables

**Table 1:** Clinical characteristics of patients enrolled at the time of MDT.

	Overall	[18F]F-Fluorocholine Guided MDT	[68Ga]Ga-PSMA-11 Guided MDT	*p* Value
Number of patients	37	11	26	
ISUP grade				
ISUP 1	8 (21.6%)	2 (18.2%)	6 (23.1%)	0.747
ISUP 2	8 (21.6%)	3 (27.3%)	5 (19.2%)	
ISUP 3	8 (21.6%)	1 (9.0%)	7 (27.0%)	
ISUP 4	8 (21.6%)	3 (27.3%)	5 (19.2%)	
ISUP 5	5 (13.6%)	2 (18.2%)	3 (11.5%)	
Primary treatment (radiotherapy vs. surgery)				
Surgery	29 (78.4%)	7 (63.6%)	22 (84.6%)	0.157
Radiotherapy (± ADT)	8 (21.6%)	4 (36.4%)	4 (15.4%)	
Previous salvage radiotherapy	29 (78.4%)	9 (81.8%)	20 (76.9%)	0.741
Time to oligometastases, months (range)	91 (2–245)	79 (2–209)	104 (2–245)	0.551
Age at MDT	73.7 ± 7.57	70.45 ± 8.9	75.08 ± 6.65	0.142
CRPC at MDT	8 (21.6%)	3 (27.3%)	5 (19.2%)	0.587
PSA pre-MDT	3.01 ± 6.34	4.19 ± 5.38	2.5 ± 6.74	0.468
Medical therapy in addition to MDT	29 (78.4%)	10 (90.9%)	19 (73.2%)	0.228
PSA nadir after MDT	0.76 ± 0.1	1.54 ± 1.83	0.43 ± 0.91	**0.018**
Time to PSA nadir, months (range)	4 (1–29)	4 (1–17)	5 (1–29)	0.545
Number of metastases treated with MDT				
1 lesion	30 (81.0%)	11 (100%)	19 (73.2%)	0.302
2 lesions	5 (13.6%)	0 (0%)	5 (19.2%)	
3 lesions	1 (2.7%)	0 (0%)	1 (3.8%)	
5 lesions	1 (2.7%)	0 (0%)	1 (3.8%)	
Site of metastases treated with MDT				
Lymph nodes	24 (64.9%)	7 (63.6%)	17 (65.4%)	0.582
Bones	11 (29.7%)	4 (36.4%)	7 (27.0%)	
Both	2 (5.4%)	0 (0%)	2 (7.6%)	
MDT total dose	34.99 ± 3.54	34.09 ± 2.02	35.37 ± 3.99	0.324
MDT biologically effective dose	112.08 ± 14.21	111 ± 15.51	112.56 ± 13.91	0.766
Progression	15 (40.5%)	9 (81.8%)	6 (23.1%)	**0.001**

Continuous and dichotomous variables were compared with the *t*-test and the chi-square test, respectively. Significant *p*-values are bolded. Abbreviations: ADT, androgen deprivation therapy; CRPC, castration-resistant prostate cancer; ISUP, International Society of Urological Pathology; MDT, metastasis-directed therapy; PSA, prostate-specific antigen.

**Table 2:** Prognostic values of clinical and imaging characteristics in the prediction of PFS.

	Univariate Analysis		Multivariate Analysis	
	HR (95% CI)	*p* Value	HR (95% CI)	*p* Value
Age	0.973 (0.914–1.036)	0.390		
ISUP grade				
ISUP 1	1.000 (Ref.)			
ISUP 2	0.694 (0.097–4.972)	0.716		
ISUP 3	0.993 (0.163–6.030)	0.994		
ISUP 4	1.474 (0.268–8.097)	0.656		
ISUP 5	1.536 (0.265–8.916)	0.632		
Primary treatment (radiotherapy vs. surgery)				
Surgery	1.000 (Ref.)			
Radiotherapy (± ADT)	2.617 (0.784–8.736)	0.143		
Previous salvage radiotherapy	0.698 (0.217–2.243)	0.546		
CRPC	6.238 (1.939–20.073)	**0.002**	4.587 (1.226–17.154)	**0.024**
PSA pre-MDT	1.005 (0.922–1.095)	0.907		
Medical therapy in addition to MDT	0.635 (0.142–2.843)	0.553		
PSA nadir after MDT	2.979 (1.882–4.717)	**<0.001**	2.937 (1.750–4.928)	**<0.001**
PET/CT imaging				
[68Ga]Ga-PSMA-11	1.000 (Ref.)			
[18F]F-Fluorocholine	3.813 (1.351–10.760)	**0.011**	2.603 (0.829–8.172)	0.100
SUVmax	1.031 (0.981–1.084)	0.232		
MTV	0.936 (0.707–1.240)	0.645		
TLG	1.002 (0.977–1.028)	0.870		
Number of metastases treated with MDT				
1 lesion	1.000 (Ref.)			
2 or more lesions	1.550 (0.532–4.518)	0.422		
Site of metastases treated with MDT				
Lymph node	1.000 (Ref.)			
Bones or both	0.926 (0.283–3.031)	0.899		
MDT total dose	0.952 (0.812–1.117)	0.546		
MDT biologically effective dose	0.993 (0.958–1.029)	0.695		

Univariate and multivariate analyses were performed with the Cox regression model. Significant *p*-values are bolded. Abbreviations: ADT, androgen deprivation therapy; CRPC, castration-resistant prostate cancer; ISUP, International Society of Urological Pathology; MDT, metastasis-directed therapy; MTV, metabolic tumor volume; PET/CT, positron emission tomography/computed tomography; PSA, prostate-specific antigen; SUVmax, maximum standardized uptake value; TLG, total lesion glycolysis.

## Data Availability

The data supporting this study’s findings are available on request from the corresponding author.
